# Diagnosis and Treatment of a Patient With Severe Combined Immunodeficiency Due to a Novel Homozygous Mutation in the IL-7Rα Chain

**DOI:** 10.3389/fimmu.2022.867837

**Published:** 2022-03-28

**Authors:** Rana Mansour, Yasmin El Bsat, Anthony Fadel, Youmna El-Orfali, Dolly Noun, Nidale Tarek, Nabil Kabbara, Miguel Abboud, Michel J. Massaad

**Affiliations:** ^1^ Department of Experimental Pathology, Immunology, and Microbiology, Faculty of Medicine, American University of Beirut, Beirut, Lebanon; ^2^ Faculty of Medicine, American University of Beirut, Beirut, Lebanon; ^3^ Division of Pediatric Hematology Oncology, Department of Pediatrics and Adolescent Medicine, American University of Beirut Medical Center, Beirut, Lebanon; ^4^ Children’s Cancer Center of Lebanon, American University of Beirut Medical Center, Beirut, Lebanon; ^5^ Division of Pediatric Hematology Oncology, Rafic Hariri University Hospital, Beirut, Lebanon; ^6^ Division of Pediatric Hematology Oncology, Centre Hospitalier du Nord, Zgharta, Lebanon; ^7^ Department of Pediatrics and Adolescent Medicine, American University of Beirut Medical Center, Beirut, Lebanon; ^8^ Research Center of Excellence in Immunity and Infections, American University of Beirut, Beirut, Lebanon; ^9^ Center for Infectious Diseases Research, American University of Beirut, Beirut, Lebanon

**Keywords:** inborn errors of immunity, severe combined immunodeficiency, IL-7Rα chain, T cell function, hematopoietic stem cell transplantation

## Abstract

The interleukin-7 receptor (IL-7R) is expressed on lymphoid cells and plays an important role in the development, homeostasis, survival, and proliferation of T cells. Bi-allelic mutations in the IL-7Rα chain abolish T cell development and function resulting in severe combined immunodeficiency disease. In this manuscript, we investigate a 1 year-old patient born to consanguineous parents, who suffered from autoimmune hemolytic anemia since birth associated with recurrent severe infections. Flow cytometric analysis of the patient’s peripheral blood demonstrated elevated numbers of B and NK cells, decreased numbers of T cells, defective thymic output, a predominance of memory T cells, and absent T cell proliferation. Next Generation Sequencing identified a novel homozygous pathogenic mutation in *IL7RA* (c.379G>A) that resulted in aberrant *IL7RA* RNA splicing and absent IL-7Rα expression. The patient was successfully transplanted using her HLA-matched relative as donor. One year after transplant, the patient is clinically stable with normal reconstitution of donor T cells that express IL-7Rα, a significant increase in the percentages of recent thymic emigrant and peripheral T cells, normalization of naïve and memory T cells, and restoration of her T cell’s proliferative response. Therefore, using genetic and functional approaches, we identified a novel deleterious mutation in IL-7Rα that results in T^-^B^+^NK^+^ phenotype, and report successful hematopoietic stem cell transplantation of the patient. This represents the first bedside-to-bench-and-back case entirely performed on a patient with severe combined immunodeficiency at the American University of Beirut Medical Center.

## Introduction

Inborn Errors of Immunity are genetic disorders that impair the development or function of immune cells leading to defective and/or uncontrolled immune responses (1). The most severe form of Inborn Errors of Immunity is severe combined immunodeficiency (SCID), a group of disorders characterized by T cell deficiency sometimes coupled with a defect in B and/or NK cells, resulting in various combinations of infections sometimes associated autoimmunity (1–3). SCID could be fatal if not diagnosed early in life, and the only curative treatment is hematopoietic stem cell transplantation (HSCT) and gene therapy in some forms (3, 4).

Interleukin (IL)-7 is secreted by stromal cells in the bone marrow and thymus, and it signals through the IL-7R, a heterodimeric complex consisting of the IL-7Rα chain in association with the common γ chain (IL-2Rγ) (5), to mediate the development and function of T and B cells in mice (6, 7), and of T cells in humans (8). Binding of IL-7 to its receptor activates several intracellular signaling pathways, resulting in the rearrangement of the T cell receptor, thymocyte maturation, proliferation, survival, and homeostasis (9–12).

Because of the role IL-7Rα plays in the development, homeostasis, and function of T cells, patients with IL-7Rα deficiency develop SCID with very few or completely absent T cells, and normal B and NK cell numbers (13, 14). Loss-of-function mutations in IL-7Rα have been widely described in the last few decades, most of them being associated with severe clinical symptoms characterized by recurrent infections and autoimmune manifestations at an early age (15, 16). Most deleterious mutations reported in *IL7RA* are single nucleotide variations resulting in amino acid changes, and are concentrated in the first 5 exons of the gene corresponding to the extracellular domain of the translated protein, with exon 2 showing the highest mutation frequency (15, 16).

In this manuscript, we describe a female patient with autoimmune hemolytic anemia and recurrent infections since birth, associated with decreased T cell numbers and function. Next generation sequencing identified a novel homozygous mutation in *IL7RA* that abolishes exons 3-4 splicing and IL-7Rα expression. The patient underwent HSCT that resulted in normal immune reconstitution and correction of her immune defect and clinical status. This work expands the spectrum of SCID due to IL-7Rα deficiency and describes the first diagnosis and treatment of a SCID patient entirely performed at the American University of Beirut Medical Center (AUBMC).

## Materials and Methods

### Subjects

Peripheral blood was obtained after securing informed consent approved by the Institutional Review Board of the AUBMC (IRB# BIO-2018-0358), and according to the Declaration of Helsinki. The study was conducted at the Immunology Research Laboratory of the Faculty of Medicine, American University of Beirut, Beirut, Lebanon.

### Flow Cytometry

Peripheral blood mononuclear cells (PBMCs) were isolated on a Ficoll-Paque PLUS gradient (GE Healthcare; IL, USA). Anti-human antibodies for surface and intracellular makers with isotype-matched controls were used for staining CD3 (UCHT1), CD4 (OKT4), CD8 (RPA-T8), CD45RA (HI100), CD45RO (UCHL1), CCR7 (G043H7), CD31 (WM59), CD16 (3G8), CD56 (5.1H11), CD127/IL-7Ra (A019D5), CD25 (BC96), CTLA-4 (L3D10), Helios (22F6), CD19 (HIB19), CD27 (M-T271), and IgD (IA6-2), all from Biolegend (CA, USA), and FOXP3 (PCH101) from eBioscience (CA, USA). Cells were analyzed on a BD FACS Aria cell sorter (BD Biosciences, NJ, USA), and analyzed with FlowJo software (Tree Star Inc.; OR, USA).

### Genetic Analysis

Genomic DNA (gDNA) was extracted from blood using the Gentra Puregene Blood Kit (Qiagen; Hilden, Germany). Custom-made primers were designed and ordered using the Ion AmpliSeq Designer (ThermoFisher Scientific, MA, USA) and used to amplify a targeted gene panel of 300 genes resulting in 6203 amplicons of intronic and exonic sequences. The products were subjected to next generation sequencing on an Ion GeneStudio S5 System (ThermoFisher Scientific), and the results were analyzed on the Ion Reporter software (ThermoFisher Scientific) using customized filters. The *IL7RA* gDNA region of interest was amplified by polymerase chain reaction (PCR) with TopTaq DNA Polymerase (Qiagen) using forward primer 5’GAGACTTGGAAGATGCAGAACTG3’ and reverse primer 5’CACACCTGGGTTTGAAGATCC3’. The amplified PCR products were purified using the QIAquick PCR purification kit (Qiagen) and subjected to Sanger sequencing. Sequences were compared to the reference sequence NM_002185.4 of *IL7RA* published at the National Centre for Biotechnology Information, and analyzed using SnapGene viewer software (GSL Biotech LLC, CA, USA).

### RNA Extraction and RT-PCR of *IL7RA* cDNA

RNA was extracted from PBMCs using TRIzol reagent (Sigma-Aldrich, MO, USA), and cDNA was synthesized using High-Capacity cDNA RT Kit (ThermoFisher Scientific). Exons 2-4 of *IL7RA* cDNA were amplified using TopTaq DNA Polymerase (Qiagen) with forward primer 5’GAGACTTGGAAGATGCAGAACTG3’ and reverse primer 5’TCCTGGCGGTAAGCTACATCG3’. The amplified cDNA bands were purified and subjected to Sanger sequencing.

### T Cell Proliferation

PBMCs were labeled with 1µM carboxyfluorescein succinimidyl ester (CFSE; Sigma-Aldrich, MO, USA) and were either left unstimulated, or T cells were stimulated with 2µg/ml phytohemagglutinin (PHA; Sigma-Aldrich) or 100 ng/ml anti-CD3 antibody (OKT3; Biolegend). Five days later, samples were stained with antibodies against CD4 and CD8, and their proliferation was analyzed by flow cytometry.

### Regulatory T Cell Staining

Treg cell staining was performed using the FOXP3 staining kit from eBioscience (CA, USA) with antibodies against the surface markers CD4 and CD25, and the intracellular markers FOXP3, CTLA-4, and Helios. Cells were analyzed by flow cytometry.

### Statistical Analysis

The student’s t-test was used to compare the differences between groups using the GraphPad PRISM software (GraphPad Software, CA, USA).

## Results

### Clinical Presentation and Immunologic Findings

**Figure 1:**
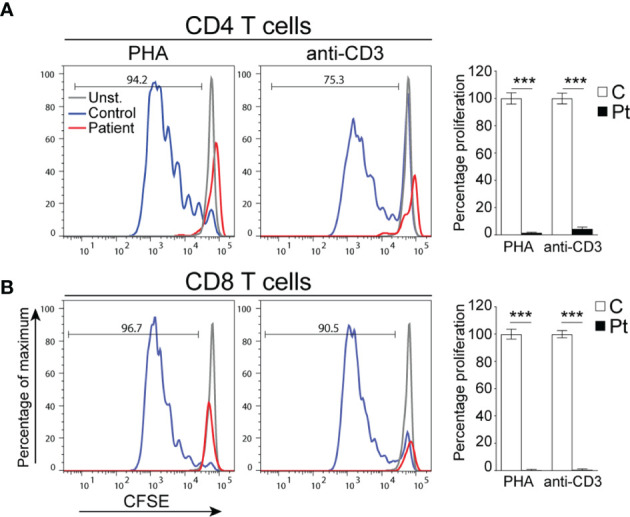
Defective T cell proliferation in PBMCs from the patient. **(A, B)** Representative histograms depicting the proliferation of CD4^+^ **(A)** and CD8^+^ **(B)** T cells. Columns and bars represent the mean ± SEM of the percentage T cell proliferation with the controls set at 100%. N = 4 controls and the patient done in 2 experiments. ***p < 0.0002.

The patient is born at term by normal vaginal delivery with no perinatal complications. She experienced recurrent upper respiratory tract infections since birth, oral thrush, bronchiolitis, and a rotavirus infection with concomitant jaundice, nausea, and vomiting for which she was hospitalized at 6 months of age. During her hospital admission, she was found to have a high reticulocyte count (18%), positive direct Coombs test, and she suffered from hemolytic anemia (hemoglobin levels 4.8 g/dl; normal range 10.8-12.6 g/dl (17)) for which she was transfused with packed red blood cells. Clinical immunophenotyping done at 7 months of age revealed decreased numbers of lymphocytes and CD3^+^, CD4^+^, and CD8^+^ T cells with normal numbers of B cells and elevated numbers of NK cells (**Table 1**). Repeated immunophenotyping up to 12 months of age demonstrated persistent anemia and severely low numbers of T cells, decreased percentages of recent thymic emigrant (RTE; CD4^+^CD45RA^+^CD31^+^) and naïve CD4^+^ and CD8^+^ (CD45RA^+^) T cells with increased percentages of memory CD4^+^CD45RO^+^ and effector memory CD8^+^CD45RA^-^CCR7^-^ T cells (**Table 1**). In addition, her few peripheral CD4^+^ and CD8^+^ T cells failed to proliferate when stimulated with anti-CD3 and PHA as compared to control T cells (**Figures 1A, B**). She had elevated numbers of NK cells, however, around 50% of her NK cells were CD56^hi^ (not shown) indicating that they have a weak effector function (22). Her B cell numbers were normal; however, because her anemia was believed to be autoimmune in nature (AIHA), she was treated with rituximab at 8 months of age to deplete her B cells. She was also treated with antibiotics, steroids, and intravenous immunoglobulin.

**Table 1:** Lymphocyte immunophenotype pre-transplant.

Age in months	7	8	10	12
Lymphocytes cells/µl	3,012 (3,400–9,000)	2,259 (3,400–9,000)	1,544 (3,400-9,000)	725 (3,400-9,000)
CD3^+^ T cells/µl	559 (1,900-5,900)	813 (1,900-5,900)	591 (1,900-5,900)	334 (1,900-5,900)
CD3^+^ T cells %	18.6 (49-76)	36.0 (49-76)	38.3 (49-76)	46.0 (49-76)
CD3^+^CD4^+^ cells/µl	181 (1,400-4,300)	203 (1,400-4,300)	213 (1,400-4,300)	81 (1,400-4,300)
CD3^+^CD4^+^ cells % of CD3^+^	6.0 (31-56)	9.0 (31-56)	36.0 (31-56)	24.3 (31-56)
CD3^+^CD8^+^ cells/µl	375 (500-1,700)	587 (500-1,700)	343 (500-1,700)	226 (500-1,700)
CD3^+^CD8^+^ cells % of CD3^+^	12.5 (12-24)	26.0 (12-24)	58.0 (12-24)	67.6 (12-24)
CD4^+^/CD8^+^ ratio	0.48	0.34	0.62	0.36
Naïve CD4^+^CD45RA^+^ % of CD4^+^	ND	1.5 (64-93)	0.82 (64-93)	16.28 (64-93)
Memory CD4^+^CD45RO^+^ % of CD4^+^	ND	98.5 (5-18)	99.2 (5-18)	83.2 (5-18)
Naïve CD8^+^CD45RA^+^ % of CD8^+^	ND	ND	9.98 (75-97)	8.92 (75-97)
Memory CD8^+^CD45RO^+^ % of CD8^+^	ND	ND	89.9 (1-8)	91.1 (1-8)
Recent thymic emigrant CD4^+^CD45RA^+^CD31^+^ % of CD4^+^	ND	9.0 (47-79)	2.33 (47-79)	5.60(47-79)
Naïve CD8^+^CD45RA^+^CCR7^+^ % of CD8^+^	ND	0.5 (34-73)	0.18 (34-73)	0.0 (34-73)
Central memory CD8^+^CD45RA^-^CCR7^+^ % of CD8^+^	ND	2.5 (3-15)	2.92 (3-15)	2.14 (3-15)
Effector memory CD8^+^CD45RA^-^CCR7^-^ % of CD8^+^	ND	92.0 (9-47)	86.4 (9-47)	87.3 (9-47)
Effector memory T_EMRA_ CD8^+^CD45RA^+^CCR7^-^ % of CD8^+^	ND	5.0 (7-25)	10.5 (7-25)	10.6 (7-25)
CD19^+^ B cells/µl	1,207 (700-2,800)	294* (700-2,800)	0.0 (400-2,900)	0.0 (400-2,900)
CD19^+^ B cells %	40.1 (15.7–34.1)	13.0 (15.7–34.1)	0.0 (13.9–28.2)	0.0 (13.9–28.2)
Naïve CD19^+^CD27^-^IgD^+^ % of CD19	ND	ND	0.0 (76.5-94.7)	0.0 (76.5-94.7)
Memory unswitched CD19^+^CD27^+^IgD^+^ % of CD19	ND	ND	0.0 (3.0-10.7)	0.0 (3.0-10.7)
Memory switched CD19^+^CD27^+^IgD^-^ % of CD19	ND	ND	0.0 (1.4-11.9)	0.0 (1.4-11.9)
CD16^+^CD56^+^ NK cells/µl	1,219 (160-950)	1,152 (160-950)	511 (160-950)	202 (160-950)
CD16^+^CD56^+^ NK cells %	40.5 (3-15)	51.0 (3-15)	33.1 (3-15)	27.9 (3-15)

The age-matched normal ranges (shown in parentheses) for lymphocyte numbers and percentages are from (18–21). ND, not determined. *The patient was treated with rituximab.

**Figure 2:**
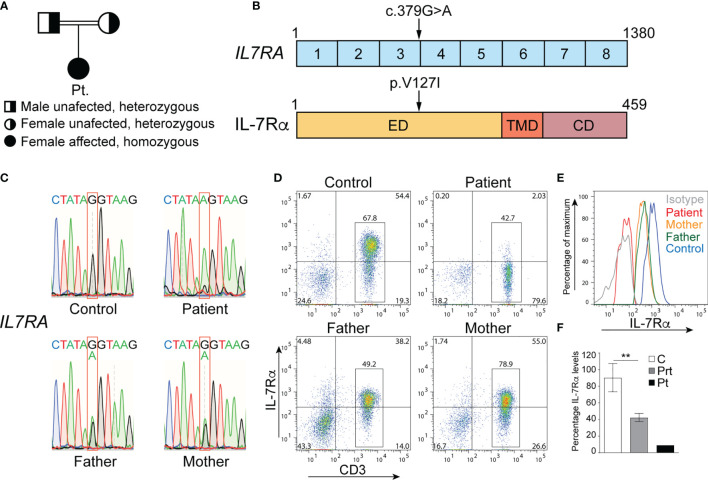
Mutation in *IL7RA* that abolishes surface protein expression. **(A)** Family pedigree. **(B)** cDNA organization of *IL7RA* and IL-7Rα protein structure showing the domains and location of the patient’s mutation indicated by the arrows. Boxes represent exons or protein domains. **(C)** Sanger sequencing chromatograms depicting the c.379G>A homozygous mutation in *IL7RA* in the patient and the heterozygous mutation in her parents, as compared to the reference sequence in the control. **(D, E)** Representative dot plot **(D)** and histogram **(E)** analysis of IL-7Rα cell surface expression on CD3^+^ T cells from the patient and her parents, as compared to a control. **(F)** Columns and bars represent the mean ± SEM of the levels of IL-7Rα expression (MFI), with the healthy controls set at 100%. N = 5 controls, the pooled parents done in 2 independent experiments, and the patient done once. **p < 0.001. ED, extracellular domain; TMD, transmembrane domain; CD, cytoplasmic domain; C, control; Prt, parent; Pt, patient.

The patient’s parents are first-degree cousins (**Figure 2A**), and her family history is notable for 4 cousins who died in infancy due to a suspected but undiagnosed immunodeficiency. Therefore, the patient’s clinical course and immune studies, as well as her family history, were all highly suggestive of a genetic disorder resulting in SCID with phenotypic and functional T cell defects.

### Genetic Analysis of the Patient

To determine the genetic etiology of the patient’s immunodeficiency, a targeted gene panel approach was used to sequence 300 genes from her gDNA, which unveiled a novel homozygous missense variant in *IL7RA*. It was the substitution of guanine (G) by adenine (A) at position 379 (c.379G>A) in exon 3 of *IL7RA*, resulting in the change of valine (V) to isoleucine (I) at position 127 (p.V127I) of the protein (**Figure 2B**). This variant was not detected in healthy (gnomAD, Exome Variant Server, and *Ensembl*) and diseased (ClinVar) individuals’ databases and was predicted to be damaging by Sorting Intolerant from Tolerant (SIFT; 0.02), polyPhen-2 (0.993), and Combined Annotation Dependent Depletion (CADD; 33). Since mutations in *IL7RA* result in SCID (8, 14, 15), we concluded that this variant might be associated with her disease.

The homozygous c.379G>A variant was confirmed by Sanger sequencing in the patient, and here parents were heterozygous for the same variant (**Figure 2C**). Importantly, the variant severely affected IL-7Rα expression as only ~2.5% of the patient’s T cells expressed IL-7Rα as compared to ~74.0% of control T cells (**Figure 2D**). Around 73% and 67% of the mother’s and father’s T cells expressed IL-7Rα, respectively (**Figure 2D**), however, the level of expression was ~50% of that observed in several controls (**Figures 2E, F**), which is consistent with the heterozygous nature of the variant they harbor. These results demonstrate that the variant is a deleterious mutation as it abolished IL-7Rα protein expression in the patient, and reduced its expression by half in the parents.

### Effect of the c.379G>A Mutation on Exon 3-Exon 4 Splicing

**Figure 3:**
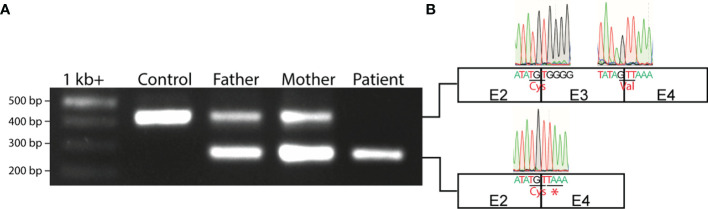
Aberrant *IL7RA* mRNA splicing in the patient. **(A)** PCR of exons 2-4 of *IL7RA* cDNA in the patient, parents, and a control. **(B)** Schematic representation of exons 2-4, and Sanger sequencing chromatograms of the exon junctions obtained from the PCR products. Shown are the last 5 nucleotides at the 5’ and 3’ ends of each exon with the respective chromatogram. Shown also are the amino acids encoded by the junctional codons, and the STOP codon that results from the alternative splicing of exon 2 to 4 in the patient. E, exon; Cys, cysteine; Val, valine; *, STOP codon.

Since G at position 379 is the terminal 3’ end nucleotide in exon 3 of *IL7RA*, substitution of G by A might influence the splice donor site of intron 3, affecting splicing of exons 3 to 4. To test this hypothesis, exons 2 to 4 of the *IL7RA* RNA/cDNA were amplified by RT-PCR, which resulted in the expected 433bp *wild type* band in the control, a smaller 275bp band in the patient, and a *wild type* and small bands in the parents (**Figure 3A**). Sequencing of the small band from the patient demonstrated aberrant splicing of exons 2-4 that bypasses exon 3, which resulted in alternative codon usage and the introduction of a TAA STOP codon a position 223-225 of the cDNA (**Figure 3B**), which terminates translation downstream of cysteine at position 74 of the protein (pC74*).

### Hematopoietic Stem Cell Transplantation

The patient underwent HSCT with myeloablative conditioning using her paternal grandmother, who shared a fully matched class I and II human leukocyte antigens (HLA), as donor. Pretransplant viral PCR for HIV, CMV, EBV, HHV6, HBV, HCV, parvovirus, adenovirus, HTLV, and toxoplasma were all negative. Pretransplant computerized tomography (CT) scan for the neck, chest, abdomen, and pelvis revealed hepatosplenomegaly.

For her conditioning she received 375 mg/m^2^ rituximab on day -7, 40 mg/m^2^/dose fludarabine IV drip on days -5, -4, -3 and -2, and 1 mg/kg/dose busulfan every 6 hours on days -5, -4, -3 and -2. Infusion of 5.18 million CD34^+^ stem cells/kg was performed when she was 12 months old without major complications. To diminish the risk of graft vs host disease (GvHD), she received 15 mg/m^2^ methotrexate on day 1, and 10 mg/m^2^ on days 3 and 6 post-transplant, as well as Cyclosporine A as of day -1 until 8 months post-transplant.

She exhibited reticulocytosis and an exacerbation of her AIHA 5 and 7 months post-transplant when she developed upper respiratory tract infections due to COVID-19 and gastroenteritis, respectively, and received packed red blood cell transfusions and 2 doses of rituximab 1 week apart 5 months post-transplant alongside monthly intravenous immunoglobulin with the last dose given 13 months post-transplant. She was maintained on a prolonged course of steroids with very slow weaning until 13 months post-transplant. She achieved 96.0% total mixed chimerism (98.0% CD3 and 97.0% CD33) 30 days post-transplant, 54.4% total mixed chimerism (99.3% CD3 and 44.8% CD33) 100 days post-transplant, and 68.0% total mixed chimerism (97.9% CD3 and 19.6% CD33) 280 days post-transplant. Her hemoglobin level is currently stable at 10-11 g/dL and her reticulocytes numbers are low. She is receiving prophylactic trimethoprim-sulfamethoxazole, fluconazole, and valacyclovir.

### Post-Transplant Immune Studies

**Figure 4:**
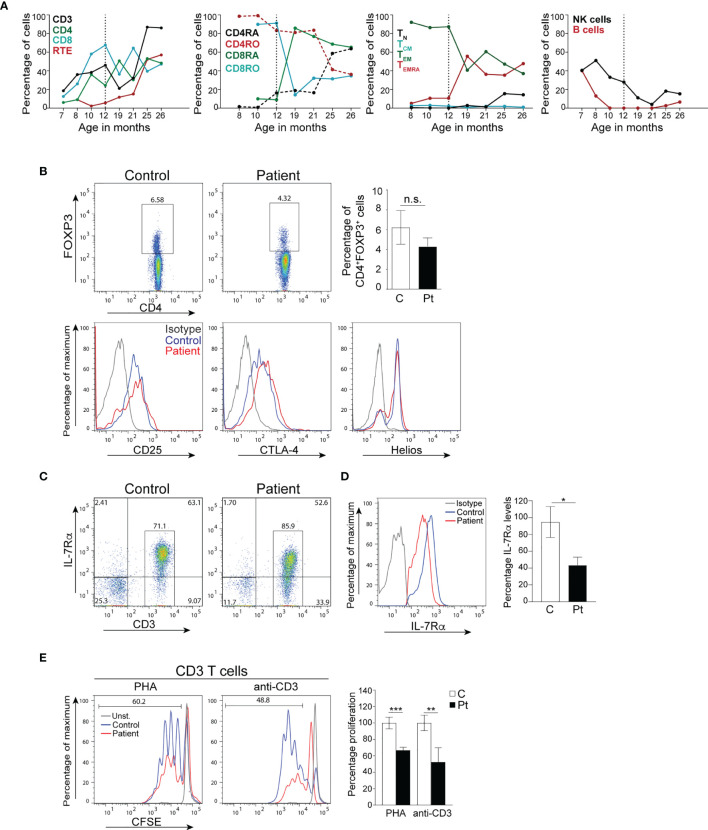
Immune reconstitution and T cell function of the patient cells. **(A)** Percentages of immune cells before and after bone marrow transplantation. The broken vertical line represents the age at transplant. **(B)** Percentages of CD4^+^FOXP3^+^ Treg cells, and expression of the effector proteins CD25, CTLA-4, and Helios by Tregs in the patient 14 months post-transplant. Columns and bars represent the mean ± SEM of CD4^+^FOXP3^+^ Treg cells. N=3 controls and the patient done in 3 independent experiments. **(C)** Representative dot plot analysis of IL-7Rα cell surface expression on donor-derived CD3^+^ T cells from the patient and a control. **(D)** Histograms represent the levels of IL-7Rα expression (MFI) on CD3^+^ T cells. Columns and bars represent the mean ± SEM of the levels of IL-7Rα expression (MFI) with the healthy controls set at 100%. N = 3 controls and the patient done 3 times. **(E)** T cell proliferation in PBMCs from the patient post-transplant. Columns and bars represent the mean ± SEM of the percentage T cell proliferation with the healthy control set at 100%. N = 3 healthy controls and the patient done in 3 independent experiments. ***p < 0.001; **p < 0.01; *p < 0.05; n.s., not significant. C, control; Pt, patient.

Immunophenotyping done at 7, 9, 13, and 14 months post-transplant demonstrated a gradual increase in the numbers of lymphocytes, CD3^+^, CD4^+^, and CD8^+^ T cells to reach the normal range for age 13 months post-transplant (**Table 2** and **Figure 4A**). In addition, the percentages of naïve T cells steadily increased with a concomitant decrease in the percentages of memory T cells. Importantly, the percentage of RTE cells increased 10-fold 13 months post-transplant indicating normal engraftment and development of T cells with normal thymic output (**Table 2** and **Figure 4A**). The percentages of naïve and central memory CD8^+^ T cells were still below of range for age, but the percentage of effector memory CD8^+^ T cells was within the range for age, and that of T_EMRA_ CD8^+^ T cells was above the range for age, indicating that the patient might still be fighting active infections (**Table 2** and **Figure 4A**). Her B cells were undetectable at 9 months post-transplant due to the rituximab treatment, but they emerged 13 months post-transplant and increased noticeably 1 month later with normal distribution of naïve and memory unswitched B cells but below the range for age of memory switched B cells (**Table 2** and **Figure 4A**). Her NK cells were still diminished at 14 months post-transplant but are expected to increase as the treatment with steroids was stopped

**Table 2:** Lymphocyte immunophenotype post-transplant.

Age in months (time post-transplant in months)	19 (7)	21 (9)	25 (13)	26 (14)
Lymphocytes cells/µl	832 (3,600-8,600)	1,794 (3,600-8,600)	2,812 (2,300-5,400)	3,690 (2,300-5,400)
CD3^+^ T cells/µl	176 (2,100-6,200)	579 (2,100-6,200)	2,444 (1,400-3,700)	3,173 (1,400-3,700)
CD3^+^ T cells %	21.1 (53-75)	32.3 (53-75)	86.9 (56-75)	86.0 (56-75)
CD3^+^CD4^+^ cells/µl	89 (1,300-3,400)	175 (1,300-3,400)	1,300 (700-2,200)	1,533 (700-2,200)
CD3^+^CD4^+^ cells % of CD3^+^	50.7 (32-51)	30.3 (32-51)	53.2 (28-47)	48.3 (28-47)
CD3^+^CD8^+^ cells/µl	64 (620-2,000)	373 (620-2,000)	965 (490-1,300)	1,498 (490-1,300)
CD3^+^CD8^+^ cells % of CD3^+^	36.4 (14-30)	64.4 (14-30)	39.5 (16-30)	47.2 (16-30)
CD4^+^/CD8^+^ ratio	1.39	0.47	1.35	1.02
Naïve CD4^+^CD45RA^+^ % of CD4^+^	18.9 (63-91)	16.5 (63-91)	58.7 (53-86)	63.7 (53-86)
Memory CD4^+^CD45RO^+^ % of CD4^+^	81.1 (7-20)	83.5 (7-20)	41.3 (9-26)	36.2 (9-26)
Naïve CD8^+^CD45RA^+^ % of CD8^+^	85.8 (71-98)	67.9 (71-98)	68.6 (69-97)	65.3 (69-97)
Memory CD8^+^CD45RO^+^ % of CD8^+^	14.2 (2-12)	32.1 (2-2)	31.4 (4-16)	34.6 (4-16)
Recent thymic emigrant CD4^+^CD45RA^+^CD31^+^ % of CD4^+^	11.7 (47-79)	15.1 (47-79)	52.3 (47-79)	57.0 (47-79)
Naïve CD8^+^CD45RA^+^CCR7^+^ % of CD8^+^	2.81 (34-73)	1.33 (34-73)	15.5 (34-73)	14.3 (34-73)
Central memory CD8^+^CD45RA^-^CCR7^+^ % of CD8^+^	0.94 (3-15)	1.91 (3-15)	1.88 (3-15)	0.92 (3-15)
Effector memory CD8^+^CD45RA^-^CCR7^-^ % of CD8^+^	40.7 (9-47)	60.5 (9-47)	47.2 (9-47)	37.0 (9-47)
Effector memory T_EMRA_ CD8^+^CD45RA^+^CCR7^-^ % of CD8^+^	55.6 (7-25)	36.3 (7-25)	35.4 (7-25)	47.7 (7-25)
CD19^+^ B cells/µl	0* (600-1,900)	0* (600-1,900)	55 (400-1,700)	242 (400-1,700)
CD19^+^ B cells %	0.0* (16.1–34.4)	0.0* (16.1–34.4)	2.6 (14.1–28.5)	6.56 (14.1–28.5)
Naïve CD19^+^CD27^-^IgD^+^ % of CD19	0.0* (68.1-89.3)	0.0* (68.1-89.3)	76.4 (54.0-88.4)	86.3 (54.0-88.4)
Memory unswitched CD19^+^CD27^+^IgD^+^ % of CD19	0.0* (4.1-13.9)	0.0* (4.1-13.9)	8.4 (2.7-19.8)	8.05 (2.7-19.8)
Memory switched CD19^+^CD27^+^IgD^-^ % of CD19	0.0* (3.9-13.6)	0.0* (3.9-13.6)	2.0 (4.7-21.2)	1.93 (4.7-21.2)
CD16^+^CD56^+^ NK cells/µl	44 (180-920)	48 (180-920)	49 (130-720)	72 (130-720)
CD16^+^CD56^+^ NK cells % of CD3^-^	11.0 (3-15)	3.94 (3-15)	18.2 (4-17)	15.4 (4-17)

The age-matched normal ranges (shown in parentheses) for lymphocyte numbers and percentages are from (18–21). *The patient received two doses of rituximab 5 months post-transplant.

Because of her AIHA, we determined the development and functional potential of her Treg cells. At 14 months post-transplant, the patient was found to have normal percentages of CD4^+^FOXP3^+^ Treg cells that expressed the effector molecules CD25, CTLA-4, and Helios (**Figure 4B**). Furthermore, ~60.1% of the patient’s T cells expressed IL-7Rα (**Figure 4C**), however, the level of IL-7Rα expression was ~50% of that observed in controls (**Figure 4D**). Importantly, the patient’s T cells proliferated effectively following stimulation with anti-CD3 and PHA, albeit to a lesser extent than that of control T cells (**Figure 4E**), which could be due to the lingering effect of the steroid treatment.

## Discussion

We describe a patient with T^-^B^+^NK^+^ SCID due to a novel pathogenic homozygous mutation in *IL7RA* that results in aberrant RNA splicing, loss of protein expression, and poor T cell production and function. The patient was successfully transplanted at the AUBMC with HLA-matched allogeneic HSCs resulting in reconstitution of her T cell development and function, and normalization of her clinical status.

The patient developed recurrent infections since birth; however, her most severe manifestation was AIHA that was probably triggered by the rotavirus gastroenteritis. Although her Treg cells were not quantified initially due to the low number of cells obtained, we suspect that they were low in numbers since the total CD4^+^ T cell numbers were low, leaving autoimmune B cells unchecked and necessitating treatment with rituximab. This immune dysregulation highlights the development of autoimmunity as one of the earliest manifestations in patients with SCID that could confuse their diagnosis (23, 24). Despite the presence of a low number of peripheral T cells in the patient, her RTEs were absent indicating defective thymic output and suggesting that her peripheral T cells likely originate from the expansion of a few T cell clones that escaped selection in the thymus or from maternal T cell engraftment, which could exacerbate her autoimmunity. Her NK cell numbers were high, however, around 50% were CD56^hi^ (not shown) indicating that they have a weak effector function, as compared to CD56^lo^ NK cells that have a higher cytolytic activity (22).

Next generation sequencing analysis of the patient’s gDNA using a targeted gene panel approach revealed a novel pathogenic homozygous c.379G>A mutation in *IL7RA*, which is consistent with the immunological phenotype of T^-^B^+^NK^+^ SCID because IL-7R*α* is essential for T cell development and function (8, 25). In addition, the vast majority (~97.5%) of the patient’s CD3^+^ T cells did not express IL-7Rα whereas only ~2.5% expressed IL-7Rα, suggesting that they might be maternal cells transferred to the patient *in utero*, as transplacentally-acquired maternal cells have been previously detected in the periphery of up to 40% of SCID patients (26). In addition, the patient’s CD3^+^ T cells failed to proliferate following stimulation with anti-CD3 antibody and PHA, providing further evidence for the role of the mutation in abolishing T cell function.

The mutated residue completely abolished splicing of exon 3 to exon 4, replacing it by splicing of exon 2 to exon 4 that resulted in an RNA that lacked exon 3, which, if translated, would encode an out-of-frame protein with premature termination of translation. This suggests that the splicing machinery is capable of splicing across exons in the *IL7RA* locus, however, it is tightly regulated to maintain proper splicing and generate a productive RNA that can be translated into the functional protein. The parents of the patient are heterozygous for the same mutation, and they expressed a *wild type* and a mutant RNA species, thus they would express half the number of IL-7Rα proteins on the surface of their T cells, which is consistent with the 50% decrease in IL-7Rα mean fluorescence intensity on their T cells. These results demonstrate that the mutation did not affect the stability of the mRNA, however, it abolished splicing of exons 3-4 that was replaced by splicing of exons 2-4, resulting in the introduction of a STOP signal downstream of cysteine at position 74 (p.C74*) of the protein rather than converting V to I at position 127. This prematurely terminated IL-7Rα translation at p.C74 and abolished IL-7Rα surface expression. Therefore, unraveling the true nature of the molecular defect is important to understand the mechanism of disease development.

The patient underwent allogeneic HSCT from her HLA-matched paternal grandmother with a myeloablative conditioning regimen. Blood samples were collected from the patient up to 14 months post-transplant to assess the engraftment and function of donor cells. Immunophenotyping analysis demonstrated an increase in the percentage of lymphocytes and T cells, normalization of the distribution of naïve and memory T cells, and a 10-fold increase in the percentage of RTEs, which indicates normal T cell development and output from the thymus. Her NK and B cell populations were still low for age but are expected to increase as the treatment with steroids was stopped 13 months post-transplant. Donor-derived Treg cells were detectable in the patient with normal expression of FOXP3. In addition, they are expected to be functional since they express high levels of the mediator molecules CD25, CTLA-4, and Helios (27–33). The appearance of donor derived functional Treg cells was concomitant with her controlled AIHA, providing *in vivo* evidence of their functional nature resulting in a positive clinical outcome.

The patient achieved complete donor chimerism in the T cell compartment and most of the patient’s T cells expressed IL-7Rα demonstrating that they are donor derived. However, the level of IL-7Rα expression on her T cells was ~50% of that observed in several controls, which suggests that the donor might have been heterozygous for the same mutation identified in the patient as she is her paternal grandmother. Importantly, T cells isolated from the patient after transplant proliferated effectively following stimulation with anti-CD3 antibody and PHA, however, their level of proliferation was lower than T cells isolated from several controls, which could be due to the lingering effect of the steroid treatment that the patient was on.

In conclusion, using genetic and functional approaches, we identified a SCID patient with a novel deleterious homozygous mutation in *IL7RA* and studied the effect of the mutation on protein expression and T cell function. Our work expands the spectrum of mutations in IL-7Rα that result in SCID, clarifies the effect of the mutation on RNA splicing, provides the genetic diagnosis needed for transplant, and determines the efficiency of donor cell reconstitution with time. (4, 34). This represents the first bedside-to-bench-and-back case entirely performed on a patient with SCID at AUBMC and paves the way for more studies in the future.

## Data Availability Statement

The original contributions presented in the study are publicly available. This data can be found here: https://www.ncbi.nlm.nih.gov/clinvar/SCV002073535.

## Ethics Statement

The studies involving human participants were reviewed and approved by Institutional Review Board of the AUBMC (IRB# BIO-2018-0358). Written informed consent to participate in this study was provided by the participants’ legal guardian/next of kin. Written informed consent was obtained from the individual(s), and minor(s)’ legal guardian/next of kin, for the publication of any potentially identifiable images or data included in this article.

## Author Contributions

MJM and RM conceived and designed the Research. DN, NT, NK, and MA contributed to the patient clinical care and transplant. MJM, RM, YB, AF, and YE-O contributed to material preparation, data collection and analysis. MJM wrote the first draft of the manuscript, and all authors commented on previous versions of the manuscript. All authors read and approved the final manuscript.

## Funding

This work was funded by a grant number 320172 from the Medical Practice Program of the American University of Beirut to MJM. The funding source was not involved in the study design, in the collection, analysis and interpretation of data, in writing the report, and in the decision to submit this article for publication.

## Conflict of Interest

The authors declare that the research was conducted in the absence of any commercial or financial relationships that could be construed as a potential conflict of interest.

## Publisher’s Note

All claims expressed in this article are solely those of the authors and do not necessarily represent those of their affiliated organizations, or those of the publisher, the editors and the reviewers. Any product that may be evaluated in this article, or claim that may be made by its manufacturer, is not guaranteed or endorsed by the publisher.
